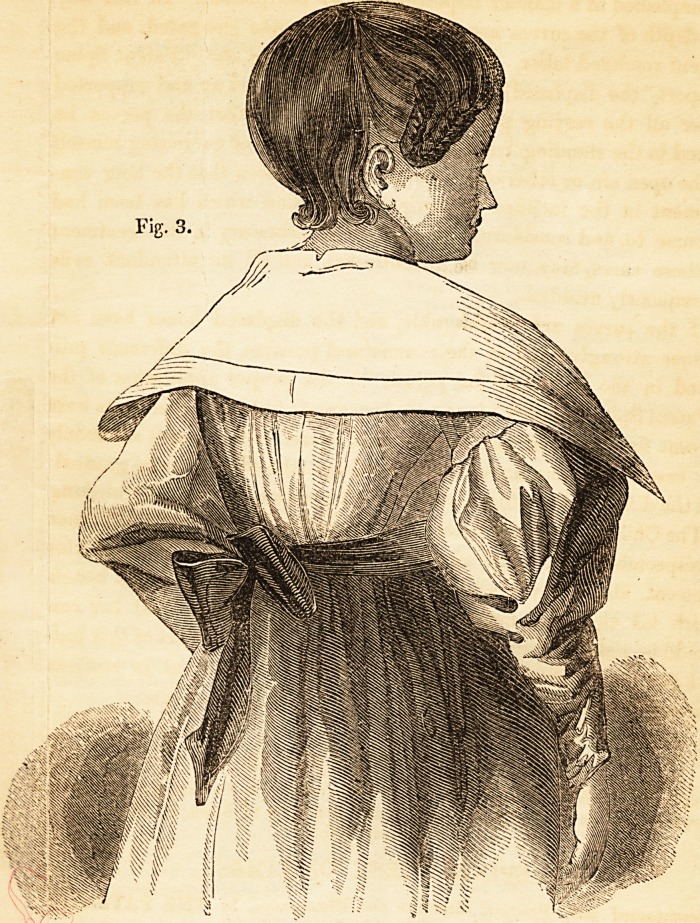# Notice of Patents Granted to Joseph Amesbury

**Published:** 1837-10

**Authors:** 


Fig. 1. represents the appearance of a little Girl 7 years [illegible] age, in whom the deforming process commenced at the age of 15 months [illegible] At the time of taking the drawing she had been under Mr. Amesbury's ca[illegible] [illegible]bout 4 months, and had greatly improved in form, strength, and health.

Fig. 2. shows the appearance of the Child a week after [illegible] application of Mr. Amesbury's "Patent Spine Support," by which she was [illegible]ediately raised *one inch and three quarters.*

Fig. 2.

Fig. 3. represents the Child as she appeared dressed, over the " Patent Spine Support," six weeks after its first application. In the course of the six weeks she has been raised *three inches and a quarter;* and consequently now stands, with the assistance of the " Patent Spine Support," *three inches and a quarter taller* than she was six weeks ago, when it was first applied; and upwards of *two inches and a half taller* than at that time, without the " Patent Spine Support," or any other aid. The circumference of the chest, over the protuberance formed by the curvature in the spine, has now diminished about three inches. The Child, before she came under Mr. Amesbury's care, was always weak and suffering, but she is now strong and healthy.

Fig. 3.

The great difference produced in the height of this Child by the application of the " Patent Spine Support," did not arise from violently stretching the spine, or any other painful process, as some might suppose, but was simply the result of lifting up the displaced bones of the back, and bringing them nearer to their natural position, which was accomplished in a manner imperceptible to the patient. In this way the depth of the curves was diminished, the spine elongated, and the patient rendered taller. By the peculiar action of the "Patent Spine Support," the displaced bones are, as it were, propped up and supported under all the varying positions of the body, whether the person be placed in the standing, lying, or any other posture, or exercising himself in the open air, or otherwise. Hence it will be seen that the long confinement in the inclined or horizontal position which has been had recourse to, and considered by many to be necessary in the treatment of these cases, may now be discontinued, and all its attendant evils consequently avoided.

If the curves are considerable, and the displaced bones have not become strongly fixed in their unnatural position, the difference produced in the height of the person by the proper application of the "Patent Spine Support" is at once very great; but where the bones have become fixed in their new position, and altered in form, as is commonly the case in persons above 20 years of age, the effect is more gradual; but the difference produced, even in such persons, is often very striking.

The Child represented in the drawings is the daughter of Mr. Turner, a respectable tradesman who has resided in Crescent Place, Burton Crescent, about 25 years; and who, in consequence of the great benefit which his child has experienced, was himself desirous that her case should be published with his name. For further particulars of this case, and for others illustrative of Mr. Amesbury's treatment in stiffness, weakness, or deformity of the Spine, Chest, and Limbs,—*see*

### NOTICE OF PATENTS

Granted to JOSEPH AMESBURY, of Burton Crescent, in the County of Middlesex, Surgeon, for certain Apparatus used in the treatment of Stiffness, Weakness, or Deformity of the

### SPINE, CHEST, OR LIMBS;

Accompanied with Practical Remarks and Illustrations, by THE PATENTEE.
Price 1*s*. 6*d*.

Published by LONGMAN and Co., Paternoster Row, London: and may be had of all Booksellers

By the same Author,

### PRACTICAL REMARKS

On the NATURE and TREATMENT of FRACTURES of the TRUNK and EXTREMITIES; being the Substance of that portion of his Lectures which relates to this Subject. Illustrated by Plates, Wood-cuts, and Cases. In 2 vols. 8vo., price 1*l*. 5*s*.

## Figures and Tables

**Fig. 1 f1:**
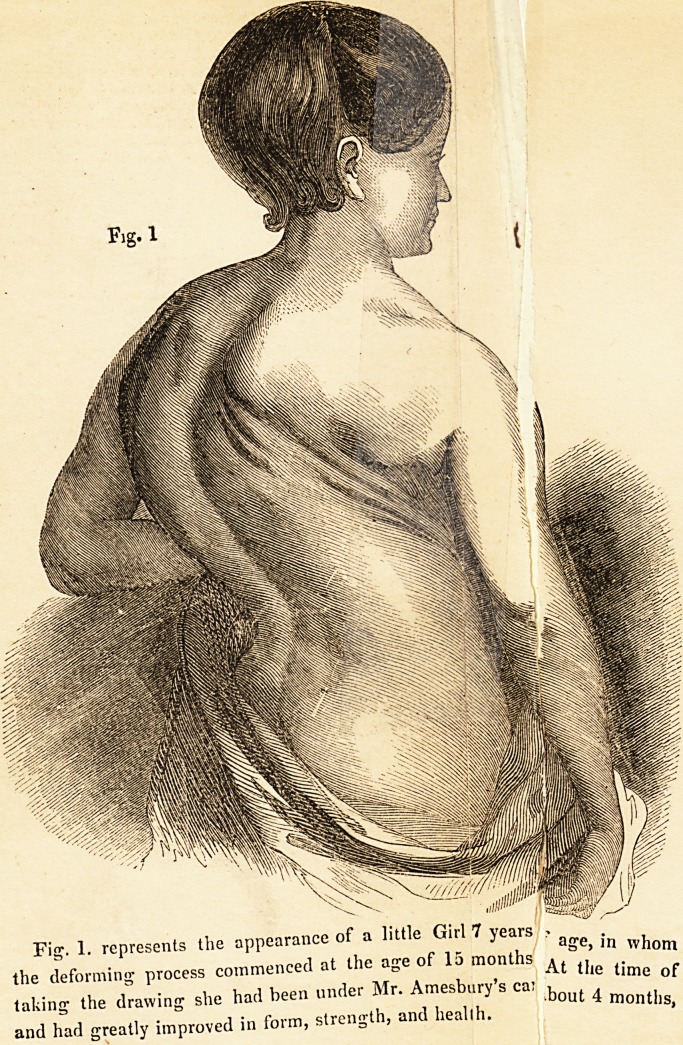


**Fig. 2. f2:**
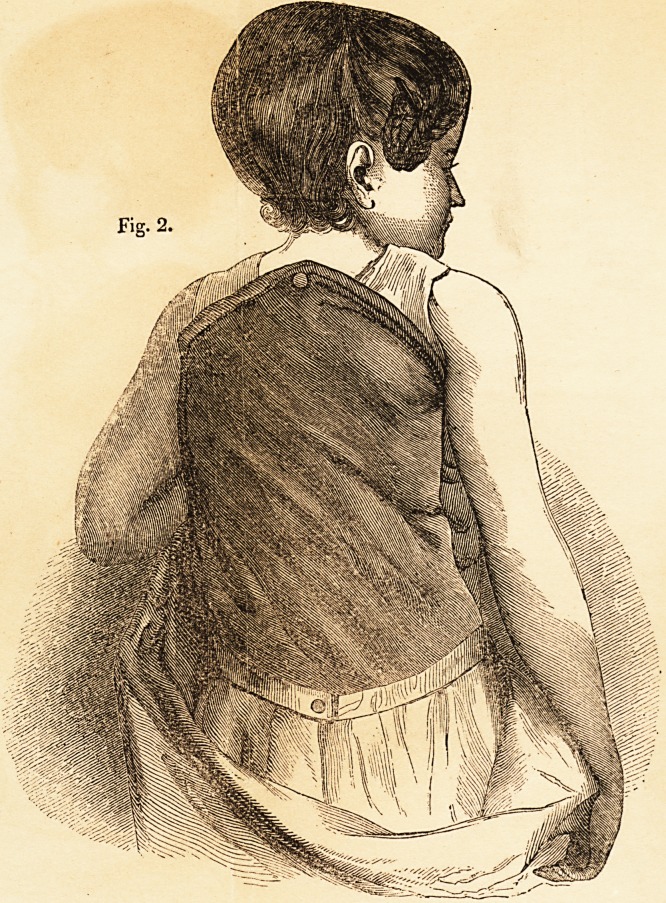


**Fig. 3. f3:**